# Sex, aging and immunity in multiple sclerosis and experimental autoimmune encephalomyelitis: An intriguing interaction

**DOI:** 10.3389/fneur.2022.1104552

**Published:** 2023-01-09

**Authors:** Marina Boziki, Paschalis Theotokis, Evangelia Kesidou, Eleni Karafoulidou, Chrystalla Konstantinou, Iliana Michailidou, Yasemin Bahar, Ayse Altintas, Nikolaos Grigoriadis

**Affiliations:** ^1^Laboratory of Experimental Neurology and Neuroimmunology and Multiple Sclerosis Center, 2nd Neurological University Department, AHEPA General Hospital of Thessaloniki, School of Medicine, Faculty of Health Sciences, Aristotle University of Thessaloniki, Thessaloniki, Greece; ^2^Hacettepe University Medical School, Ankara, Turkey; ^3^School of Medicine, Koç University, Istanbul, Turkey

**Keywords:** sex, relapsing-remitting multiple sclerosis, progressive multiple sclerosis, experimental autoimmune encephalomyelitis, aging

## Abstract

Multiple sclerosis (MS) is a chronic inflammatory disease of the central nervous system (CNS) with a profound neurodegenerative component early in the disease pathogenesis. Age is a factor with a well-described effect on the primary disease phenotype, namely, the relapsing-remitting vs. the primary progressive disease. Moreover, aging is a prominent factor contributing to the transition from relapsing-remitting MS (RRMS) to secondary progressive disease. However, sex also seems to, at least in part, dictate disease phenotype and evolution, as evidenced in humans and in animal models of the disease. Sex-specific gene expression profiles have recently elucidated an association with differential immunological signatures in the context of experimental disease. This review aims to summarize current knowledge stemming from experimental autoimmune encephalomyelitis (EAE) models regarding the effects of sex, either independently or as a factor combined with aging, on disease phenotype, with relevance to the immune system and the CNS.

## 1. Introduction

Multiple sclerosis (MS) is an autoimmune disease of the central nervous system (CNS) with its neurodegenerative component being increasingly recognized and studied over the last years (1). Similar to the majority of autoimmune diseases (2), a female predilection is evident, based on large epidemiological data from several countries (3). Moreover, MS is a highly heterogeneous disease with a very diverse range of clinical manifestations, radiology, course, and prognosis. In addition to sex, age of onset, as well as aging are also considered significant factors for the overall disease course and prognosis. Aging is defined as the biological processes that occur over time affecting all organs on a tissue and cellular level. Aging is characterized by profound alterations in an organism's physiology and homeostasis, thus affecting several systems and functions, such as the metabolism, endocrine function, and the immune system (4). Recent advances with respect to “omic” technologies have allowed detailed fingerprinting of disease phenotypes, as well as of the underlying pathological processes, on a tissue and cellular level. It is prominent that sex variations account for specific differences in aging processes, thus being responsible for sex disparities in disease phenotype and progression (5).

The sex variation and aging in CNS autoimmunity have been extensively studied in experimental models of MS, prominently in the rodent model of experimental autoimmune encephalomyelitis (EAE). Although not entirely similar to MS, EAE recapitulates several aspects of disease pathology and is considered the most widely used experimental model for MS (6, 7). In the induced model of EAE, CNS autoimmunity is triggered by active immunization of rats or mice with an encephalitogen protein and/or peptide factor, together with an adjuvant. EAE is typically induced in female laboratory animals of young age, specifically 4–6 weeks of age, in order to investigate sex and age-related differences that would induce significant heterogeneity in the model. Moreover, experimental evidence highlights alterations in disease processes under various effects of sex and age. This review aims to summarize current knowledge stemming from EAE models regarding the effects of sex, either independently or as a factor combined with aging, on disease phenotype, with particular relevance to the immune system and the CNS.

## 2. Sex and aging in MS

### 2.1. Sex and MS phenotype: Clinical evidence

In most epidemiological studies addressing the incidence and prevalence of MS, there is a consistently higher predilection for women in relation to MS frequency, varying from 2:1 to 3:1 with respect to the female-to-male ratio (8). This pattern appears universal and is not affected by latitude within the countries studied (3). Moreover, the female-to-male ratio for MS appears to increase over the last decades in several nationwide studies, an observation possibly linked with alterations in environmental factors (9–15). Evidence on the effect of environmental factors on MS incidence has long been described in epidemiological studies (16), with Epstein–Barr virus suspected to be related to the events preceding the disease onset for a long time. However, knowledge of the exact mechanisms by which EBV infection may contribute to MS incidence is lacking. Epidemiological evidence has recently identified EBV infection as a trigger of MS (17). Interestingly, the expression levels of genetic risk loci linked with MS were shown to differ between males and females, possibly due to epigenetic modifications (18). Furthermore, in EBV-infected B cells *in vitro*, the expression levels of estradiol receptors were shown to correlate with EBV infection traits, such as EBV latency III genes, thus providing evidence toward the sex-associated pathways of pathogenicity in MS and EBV-related pathology (18).

In addition to the effects of sex on MS incidence, extensive research focuses on whether sex is related to different clinical and/or radiological outcomes, as well as disease prognosis. The annual relapse rate has been reported to be higher for female patients, although a profound positive association between increased annual relapse rate and younger age has also been described (19–21). These findings indicate a higher inflammatory component for female patients with relapsing-remitting MS. However, these findings do not coincide with faster disability accumulation for female patients as the male sex is reportedly linked with faster disability outcome deterioration over the disease course than the female sex (21, 22). Of note, these differences were balanced for men and women after the age of 50 years, thus indicating sex-hormone-dependent mechanisms in relation to disease course (21). A beneficial effect of estrogens on MS outcomes has been described in studies addressing alterations in the disease course in menopausal patients with MS compared with patients before menopause onset (23, 24). Moreover, disability accumulation reportedly occurs faster in female patients with later-onset MS, i.e., >40 years old at disease onset, than in male patients with MS, and this sex-associated difference has been related to differential gene expression profiles in men and women (25). Conversely, male patients with relapsing-remitting MS have been correlated with faster conversion toward progressive disease, namely, secondary progressive MS (SPMS) (26, 27). This observation has been further supported by transcriptomic profile analysis in female and male patients with MS, revealing sex-specific molecular mechanisms in disease evolution (25, 28).

### 2.2. Aging and MS phenotype: Clinical evidence

In addition to the chronological age, defined by the date of birth, biological age is depicted by the relative assessment of molecular markers linked with basic biological processes of aging. Over the last years, through the development of multi-omics, defined as an integrative fingerprinting analysis approach that encompasses datasets generated from genomics, epigenomics, transcriptomics, proteomics, and metabolomics (29), considerable knowledge has been acquired in the field of systems immunology and the complex interactions with other basic biological mechanisms, such as aging. Aging represents a paradox of immunodeficiency and inflammation (inflammaging), with profound implications for autoimmunity. Upon aging processes, genetic and epigenetic changes confer alterations in pathways of innate and acquired immunity, thus differentially shaping the antigen receptor repertoires and dysregulating the complex interactions between cellular and molecular components of immune response (30). More specifically, several basic biological mechanisms are considered the pillars underlying aging processes, including but not restricted to, for instance, genomic instability, epigenetic changes, cellular senescence, and changes in intercellular communication (30). Moreover, aging processes result in age-related phenotypes with implications for the overall function and wellbeing, such as loss of muscle mass, age-related diseases, and frailty (30). Immune alterations in the elderly and extremely old or over-aged (age >85 years) have long been studied and recognized as alterations in the circulating immune cell types and the lymphocyte subpopulations, as well as diminished responses against the antigen (25). Additionally, in aging organisms, deficient clearance of senescent cells results in the accumulation of cellular and molecular debris, with further harmful effects on tissue homeostasis (30).

Immune alterations in the context of aging have been implicated in MS phenotype with advancing age, as well as the several comorbidities that accumulate in older patients with MS. Inflammaging is characterized by low-grade, chronic, and systemic inflammation in the elderly and is associated with the predominance of pro-inflammatory phenotypes in cellular components of the innate and adaptive immunity, the proinflammatory cytokine and chemokine production, and the expansion of senescent cellular phenotypes (5). Nevertheless, individual aging and inflammaging mechanisms appear to be diverse among individuals (31), and this heterogeneity may account for the varying burden of inflammaging in autoimmune diseases of the elderly and the age-related phenotype alterations in MS (32, 33).

In MS, neuroinflammation and neurodegeneration are pathological processes that coexist in the CNS and their relative contribution across the disease course is a primary factor for the disease phenotype, assessed by clinical outcomes that depict relapse activity and/or disability accumulation (34). The age of disease onset is a determinant factor for the disease phenotype. Patients with younger onset ages develop a relapsing-remitting form with a high neuroinflammatory pathological component, whereas patients with older onset ages at onset more frequently display progressive disease. For patients of older age at onset, progression is present either from the onset, thus signifying the primary progressive disease form (PPMS), or appears faster in the disease course, due to a more rapid conversion from RRMS to SPMS, than patients of younger age at onset (35). Thus, the disease phenotype and the transition from RRMS toward SPMS appear to be primarily an age-dependent phenomenon (36). Moreover, age progression signifies a reduction in the annualized relapse rate, most likely linked to a reduction in the effectiveness of the disease-modifying treatments (DMTs), as these factors primarily target the neuroimmune aspects of disease activity (16, 37). In line with these epidemiological observations, clinical MRI data confirm accelerated cortical atrophy and brain volume loss in patients with progressive disease compared with RRMS, and these MRI alterations are enhanced with advanced age (38) and correlate with clinical outcomes of disability accumulation (39). Other biological markers known to correlate with age, such as the serum neurofilament light chain, also appear to correspond, at least in part, to clinical and MRI markers of increased MS pathology, and their concentration increases over time in patients with MS (40, 41).

## 3. Sex and EAE phenotypes

Experimental autoimmune encephalomyelitis is one of the most widely used animal models for MS, where neuropathological mechanisms and evaluation of miscellaneous therapeutic compounds can be studied (42–44). In addition to EAE, Theiler's murine encephalomyelitis virus infection and chemically induced demyelination (cuprizone and lysolecithin) reflect axonal impairment and remyelination processes in MS, respectively. Although no experimental model has established all aspects of human MS, EAE is considered to be the most suitable (45). There are several EAE models, and each one can bring to life different hallmarks of the disease (46). EAE models fundamentally mimic the immune aspect of MS; active lesions, CNS infiltration of peripheral macrophages, relapsing-remitting events, and microglia and astroglia accumulation. Besides neuroinflammation, EAE progression can resemble chronicity and include neurodegeneration, axonal loss, demyelination, and even remyelination to a lesser extent. This dual nature of EAE can potentially become a tripartite, an event trifecta considering one more key player, which is the sex. Sex-dependent differences are quite prominent, as seen through experimental approaches primarily in rodents, and will be further reviewed in the following sections.

### 3.1. EAE models recapitulate different aspects of the MS disease pathology

Experimental autoimmune encephalomyelitis can be induced either passively or actively. Passive induction of EAE through adoptive transfer of activated encephalitogenic T-cell clones allows the study of differentiated Th1 or Th17 populations and their cell trafficking in the recipient mice (47). Active immunization and induction of EAE are achieved by the administration of encephalitogenic peptides such as proteolipid protein (PLP), myelin oligodendrocyte glycoprotein (MOG), myelin basic protein (MBP), and spinal cord homogenate. In most cases, pertussis toxin is co-administered to increase both the incidence and severity of the disease. Pathogenesis of EAE exhibits ascending paralysis, and the typical assessment tool is a clinical score resembling the Expanded Disability Status Scale score in human disease. Despite the similarities, each model can provide different insights into the MS-like progression and pathogenesis (48).

One of the first EAE models used was the active immunization of Swiss Jim Lambert (SJL) mice with PLP139–151, which fairly recapitulates a relapsing/remitting disease phenotype (49). Moreover, as occurs in humans, it is intriguing that the establishment of EAE is more severe in female mice. The initial phase begins on day 10 post-immunization, culminating around day 25 and the relapse is evident on day 40. Relapse arises from the expansion of epitope spreading, in which, due to secondary endogenous peptides, reactive T cells emerge as a consequence of the initial phase of myelin destruction. Remission is associated with a temporary loss of inflammatory cells. This particular model is used to study autoimmune T-cell-mediated responses, the compromise of the blood–brain barrier (BBB), relapse mechanisms in epitope spreading, antibody-mediated demyelination, and the evaluation of many anti-inflammatory therapeutic compounds for RRMS (50).

MOG35-55 EAE in C57BL/6 mice initiates a chronic–progressive form of EAE (51). On the contrary, if Biozzi ABH mice are immunized with the same encephalitogenic peptide, animals will display a more RRMS clinical outcome (52). In either case, this phenomenon implies that MOG peptides are unique in that they trigger an encephalitogenic T-cell response and a demyelinating autoantibody-mediated response to certain mouse strains (53). Moreover, mice exhibit microglial and astrocyte activation both in white and gray matter, as well as neuronal and synaptic loss in gray matter. Axonal damages contribute to a self-sustained chronic neurodegenerative process due to the presence of outgrowth inhibitory factors (54), which is established even in the absence of continued peripheral cell infiltration. In addition to paw paralysis, mice show evidence of CNS demyelination both in the spinal cord and the brain. This model has been used to investigate neurodegenerative mechanisms, axonal loss, T-cell priming, Th1/Th17 CD4+ T-cell-mediated CNS damage, and T-cell self-tolerance. Moreover, it is considered the most suitable for compound profiling and preclinical evaluation of cellular therapies and restorative agents (55).

The least employed model is the spontaneous EAE model, which offers the advantage of studying autoimmune mechanisms developing in a genetically controlled background and eliminating the effect of exogenous manipulations (56). Numerous strains are suitable such as C57Bl/6, SJL, and B10.PL, and some are even genetically modified for specific susceptibility factors such as humanized Tg(HLA-DR2) and humanized Tg(HLA A3) (57). Spontaneous EAE models exhibit paralysis, optic neuritis, ataxia, and present sparse levels of progression. They are used to study spontaneous T-cell activation mechanisms and innate immune mechanisms. Spontaneous EAE models are excellent tools to study B-cell responses in EAE.

### 3.2. Sex-specific aspects of the immune system: Lessons from EAE

There is accumulating evidence that males and females exhibit different immunological responses throughout life, whereas others are only present after puberty and before reproductive senescence, suggesting that both genes and hormones are involved. EAE can be a useful tool to unravel those sex-specific differences in immune responses (58). Notably, different strains display different susceptibility to EAE. For example, C57Bl/6, SJL, ASW, and NZW mice demonstrate an increased tendency for EAE in females than males, but B10.PL and PL/J are more prone to diseases in males than females (59). Moreover, androgens such as testosterone are considered an ameliorative factor in SJL/J EAE, and gonadectomy of male mice makes them more vulnerable to EAE severity (60). On the contrary, *in vivo* administration of testosterone ameliorates EAE severity and favors T helper 2 proliferation in an MBP immunization model (61), whereas administration of exogenous testosterone on female splenocytes *in vitro* minimizes the ratio of IFN-γ:IL-10 (62). Taken together, experimental data denote that female mice are prone to worsened EAE with low levels of testosterone being a potential factor. A more detailed view of the specific aspects of this sexual dimorphism is presented in the following sections.

#### 3.2.1. Chromosome-based aspects

The relative contribution of sex and chromosomes to immunological processes can be investigated with the four-core genotype (FCG) mouse model (63). The FCG model provides valuable information regarding genital determination that can be separated from the inheritance of the Y chromosome (64). Double transgenic mice can have four different genotypes and can be either XX gonadal males or females and XY gonadal males or females, respectively. This is due to Sry knockout of the Y chromosome (YSryKO), which can be ectopically expressed on chromosome 3 (Tg-Sry) (65). In the absence of Sry, animals undergo the female hormonal pathway and can be hormonally and chromosomally female (XX) or hormonally female and chromosomally male (XYSryKO). On the contrary, in the presence of Sry animals become hormonally male and can be hormonally and chromosomally male (Tg-Sry XYSryKO) or hormonally male and chromosomally female (Tg-Sry XX).

A complementary model called the XY^*^ model, in which a male produces XX, XO, XY, and XXY gametes, can be used to identify the precise mechanism underlying a sex difference observed by the FCG model (66). Hormonal influences and sexual chromosomal interactions can sometimes counteract each other's effects, whereas this method has been occasionally used to identify particular X or Y genes that either worsen or protect against a disease (67). For example, double transgenic FCG mice have revealed sex and chromosome-related findings upon active immunization in EAE on the SJL/J background. In this scenario, XX SJL/J mice and Tg-Sry XX, both chromosomally female mice, develop EAE of greater severity than chromosomally male mice XYSryKO and Tg-Sry XYSryKO. In addition, passive induction of EAE with adoptive transfer of XX T-cells triggered EAE of greater severity than XYSryKO T-cells. These findings were indicative of the T-cell compartment, which makes female mice prone to develop EAE of greater severity (68).

Different series of experiments explored neurodegeneration, and severe clinical disease was associated with the expression of the X gene Toll-like receptor 7 (TLR7), known to induce neuronal damage. These findings may indicate differential expression of TLR7 in the male and female CNS and could explain the increased susceptibility in women (69). This also could explain why female SJL/J mice develop EAE of higher severeness in contrast to males upon adoptive transfer (68). Parental imprinting, an effect in which a single locus will entirely shape one's phenotype, although two alleles are inherited, has also been studied under such circumstances (70). Teuscher and colleagues crossed XX C57BL6/J female mice with B6 males that carried Y chromosome variants known to cause susceptibility to autoimmune diseases and revealed that the EAE severeness of female progeny was dependent on the Y chromosome of their male siblings. Moreover, susceptibility in several clinical subtypes of EAE in both males and females was related to autosomal EAE loci on chromosome 13 (71). This locus was positively linked to susceptibility of monophasic remitting/non-relapsing EAE in males but not females; therefore, it is possible that endogenous androgens may be EAE-protective in a given strain depending on the allele 13 inheritance (72).

#### 3.2.2. Hormone-based aspects

An intriguing topic is the effect of male vs. female hormones in immune responses and how they affect the establishment or even the progress of EAE as most terminally differentiated immune cells express sex hormone receptors (73). Estrogens include estrone (E1), estradiol (E2), and estriol (E3) – produced only during pregnancy – and they act through estrogen receptor alpha (ERα) and estrogen receptor beta (ERβ). Androgens, in contrast, bind to androgen receptors (AR), respectively, and these were found in neutrophils, macrophages, B cells, and T cells. Studies revealed that CD4 T helper cells express more ERα than Erβ, and CD8 T cells and monocytes express low amounts of both ERs. On the contrary, B cells express significantly increased amounts of ERβ than Erα, and antigen-presenting cells express both ERα and ERβ (74).

As mentioned earlier, both androgens and estrogens can modulate EAE progression and severity; however, estrogens seem to operate in a more diverse way. The leading hypothesis relies on a threshold effect through which sex-specific hormones can reflect their dynamic through a protective vs. harmful equilibrium. For estrogens specifically, an accumulating body of evidence implies the negative contribution in the thymus and T-cell maturation (75, 76). This peculiar action may be mediated by distinct ER types, affecting the T-cell maturation pathway, either directly or indirectly. Estrogens can directly influence the developing T cells or indirectly can affect thymic epithelial cells to inhibit secretion of pivotal elements for T cells or generation of signaling important for thymocyte survival (75). Moreover, E2 is far more vigorous than testosterone in accelerating thymic atrophy. Estrogens, at least partly, can also induce thymocyte apoptosis and finally cause thymic atrophy (76, 77). This detrimental effect of estrogens has been mediated again *via* both ER receptors (78, 79) and GPR30-mediated mechanisms (77).

Continuing with the deleterious effect of estrogens, a study on thymic involution in pregnant mice implies that increased estrogen and progesterone levels during this period affect the proliferation of T-cell repertoire (80). More specifically, pregnancy does not directly affect thymocyte precursor populations in the bone marrow, but instead triggers a detrimental loss of early thymic progenitors in the thymus as early as day 12.5 of pregnancy. The similarities between estrogen-mediated involution and pregnancy-mediated involution suggest that estrogen is a pivotal regulator of loss of thymocyte cellularity during pregnancy, and probably functions primarily by reducing thymocyte proliferation (80). The same research team showed an injection of 17β-estradiol into mice causes excess loss of early thymocyte precursors and inhibits the proliferation of developing thymocytes (81). In addition, exogenous E2 supply may minimize levels of CD4+CD25+FoxP3+ T regulatory cells (Tregs) that are responsible for maintaining immunosurveillance, and loss of function of foxp3 gene in those cell populations is associated with immune-mediated inflammatory lesions (82). Collectively, the aforementioned data support an increased incidence of EAE in female rodents; however, for a complete understanding of the complex estrogen effects on T-cell development, miscellaneous details are still lacking.

In contrast, protective effects of estrogens have also been revealed and numerous studies show that estrogen treatment (with estriol and estradiol) ameliorates both active and adoptive EAE in different mice strains such as SJL, C57BL/6, B10.PL, and B10.RIII (83–89). Furthermore, estrogen treatment has been associated with reduced chemokine levels in the CNS of mice with EAE and affects the expression of matrix matalloprotease-9 (MMP-9), each leading to impaired recruitment of cells to the CNS (87, 90). Interestingly, estrogens modulate astrocytic response to injury (91) and exhibit anti-inflammatory effects on microglial activation (92). Estradiol administration may also mediate neuroprotective action in both white and gray matter pathology in spinal cords of mice with EAE by downregulation of microglial/monocyte (Mac 3+) cells on gray matter (93). Thus, apart from the anti-inflammatory effects of peripheral immune cells, estradiol treatment also suppresses CNS white matter inflammation and demyelination.

Estrogens can also regulate the equilibrium of anti-inflammatory T- and B-cell production in favor of homeostatic maintenance. It has been shown that they can promote the production of Tregs by upregulating the expression of FoxP3 (94, 95), an X-chromosome gene whose expression is higher in males than in females (96), along with other Treg subsets such as B regulatory cells (Bregs), CD8+ CD122+ Treg cells, and CD11b+ CD206+ ARG-1+ M2 such as macrophages (82). Furthermore, estrogens can directly modulate adaptive immune responses of B cells and T cells mediating changes in lymphopoiesis and can more specifically affect the expression of autoimmune regulator (AIRE) protein, which is a major regulator in the thymic expression of self-antigens (97). Finally, an interesting study in pregnant mice revealed that increased levels of E2 during pregnancy ameliorate B-cell number and activity of B lymphocyte precursors in the bone marrow, a finding that can imply that estrogen can act as a pivotal regulator of B-cell lymphopoiesis (98).

Of utmost importance is that these sex-specific differences in hormone levels can be reflected in their receptor expression, which, in turn, can affect treatment responses in EAE. For instance, an ERα-dependent increased dose requirement for the effective treatment of female vs. male mice using partial MHC (pMHC) class II constructs in chronic EAE was found (99). Results suggested that the divergence in effective dose for the treatment of chronic EAE with DRα1- mMOG-35-55 is sex dependent, and the DRα1-mMOG-35-55 treatment efficacy of female mice depends on estrogen signaling through ERα. In addition, DRα1-mMOG-35-55 treatment can upregulate the levels of CD206+ CD11b+ M2-like macrophages/microglia found in spinal cord with a significant reduction in the expression of proinflammatory genes and enhancement of genes involved in neurosurvival and regeneration (100). A similar experiment has demonstrated that RTL401, an I-As /PLP-139–151 construct, could reverse clinical disease and ongoing CNS damage in male SJL/J mice with relapsing-remitting EAE even when administered on day 20 post-disease induction (101). Elucidation of sex-specific differences may lead to insights into the evaluation of which elements are hormonally regulated and may allow design of better therapies for both sexes.

Finally, the mechanisms underlying the protective effects of androgens have also been studied. Testosterone treatment ameliorates EAE severity *in vitro* mediating its effect through Th2 bias. More specifically, androgen-treated T-cell cultures secrete lower amount of IFN-γ than IL-10 relative to untreated controls (102). These results are in total agreement with experiments in T-cell cultures from PLP139-151 immunized mice that were stimulated with PLP139–151 peptides and treated with 5a-dihydrotestosterone (DHT). Results revealed increased IL-10 and decreased IFN-g production in cultures treated with DHT (62). Similar results with increase in IL-10 and decrease in IFN-g were observed *in vivo* too (61). Ultimately, testosterone was associated with improved structure and function of dendrites and synapses in the cerebral cortex in cognitive neurodegenerative models (103), findings quite encouraging considering that synaptic stripping and loss are observed in MS cortex (104).

#### 3.2.3. Other sex-specific aspects

Sex hormones can also indirectly regulate CNS functions. For example, estrogen reduces BBB inflammation through annexin A1 (ANXA1), intercellular adhesion molecule 1 (ICAM-1), and vascular cell adhesion molecule-1 (VCAM-1) (105). Maggioli and colleagues showed that estrogen binding to ERs promotes ANXA1 phosphorylation, which efficiently recruits formyl peptide receptor 2 (FPR2) and stabilizes BBB tight junctions *via* actin reorganization (106). Another molecule implicated in BBB integrity is sphingosine-1-phosphate receptor 2 (S1PR2), while being validated as a sex-specific mechanism that disproportionately affects women. S1PR2 was highly expressed in female EAE mice relative to male EAE mice or healthy controls, directly correlated to disorganized BBB tight junctions (105). Furthermore, extracellular matrix composition in EAE mouse cortical tissue was modified, with an increased stiffness of the female cohort, showcasing again sexual dimorphism in specific collagen genes (107). Finally, brain energy metabolism is particularly interconnected to immunity. A key molecule that is highly expressed in male T cells is peroxisome proliferator-activated receptor alpha (PPARa), providing an advantage to males over the deleterious effects of EAE (108, 109).

It is already established that C57BL/6 female mice after active immunization with MOG35-55 peptide exhibit increased spinal cord infiltration and demyelination than males. On the contrary, males demonstrate increased inflammatory response but elevated regulatory cell types than females (110). Experiments on MOG EAE mice after engraftment of microglia-like cells demonstrate increased expression of CNS inflammatory factors in female but not in male mice accompanied by upregulated major histocompatibility complex class II expression of infiltrating Ly6C-hi monocytes during EAE peak and cytokine production in the female CNS (111). Complement activation *via* astrocyte compartment may also be a key player in chronic EAE establishment. More specifically, striking upregulation of C3 expressing marker for astrocytes (A1 phenotype) was found in EAE females with increased axonal loss, whereas EAE males expressed the THBS1 astrocyte marker (A2 phenotype) manifesting as a neuroprotective potential (112).

#### 3.2.4. Sex-specific aspects in relation to aging: Epigenetic modifications

The low concordance rates of MS in monozygotic twins (25), the missing heritability (27, 31), the parent-of-origin effect on disease transmission (30), and the implication of environmental factors (32, 33) in the pathogenesis suggest an effect of epigenetic mechanisms on the predisposition to MS. Changes in the levels of DNA methylation (31) and histone acetylation (15), and altered expression of micro (mi)RNAs (16) were identified in patients with MS. The possible impact of such modifications on myelin and immune gene expression is a topic of interest as certain epigenetic signatures were functionally related to oligodendrocytes (17), B (29, 34), and T cells (35, 37).

The age and sex are two variables associated with epigenetic modifications in MS and related experimental models. In cuprizone-induced demyelination, chronological age influences the mechanisms of histone acetylation affecting the intrinsic capacity of oligodendrocyte progenitors to remyelinate (36). In MS, chronological age was negatively correlated with methylation at the VMP1/MIR21 locus and expression levels of miR-21 in CD4+ T cells (16). Estimation of biological age based on DNA methylation showed accelerated aging of glial cells in patients with MS compared with controls (38). Notably, females had a reduced level of global methylation in leukocytes (39) and often showed dissimilar ratio between MS cases and controls in genome-wide DNA methylation studies [reviewed in Zheleznyakova et al. (41)] than men. Overall, these data suggest that the age and sex should be accounted for when analyzing epigenetics data and further studied experimentally for their role in MS in relation to epigenetic modifications.

## 4. Sex and aging-related neuroinflammation

A wide range of molecular pathways cooperate to buffer homeostasis as we age, which inevitably results in a functional decline and illness trajectory. Although the hypothalamus, which controls reproductive function, was once the only part of the brain thought to be responsive to sex hormones, it is now widely acknowledged that the whole brain is both a target and a source of sex hormones (113). Primarily in the adult brain, an arsenal of sex hormones has a variety of protective and antioxidant effects that ensures neural cell protection and prosperity. However, sex-based hormonal decline can be spotted in both sexes; ovarian hormones are lost very quickly after menopause, whereas testosterone dramatically diminish in an aging organism, findings that could potentially lead to age-related neurodegenerative diseases such as PPMS (114) and Alzheimer's disease (AD) (115). This reproductive senescence effect will be further discussed on the basis of the key immune cell regulator of the CNS, microglia, which is primarily affected by aging and adversely orchestrate acute and chronic reactions in EAE.

### 4.1. Do males and females age differently? Microglia implication for the neuroimmune axis

As mentioned earlier, a wide variety of profound alterations occur between sexes in the immunocompromised brain. Microglia modulate the microenvironment in physiological conditions maintaining homeostasis toward a healthy brain. Impaired surveillance of this resident immune population has been partly held accountable for aging in both sexes (116). More specifically, it is the neurodegenerative senescent state of microglia, and to a somewhat extent astroglia, that drives this sex-specific motif (117). However, as microglia constitute the sole immunocompetent cells of the CNS and these cells are dysregulated by aging, current research is being focused on the key sex differences in multiple levels such as the hormonal (118–120), chromosomal (121, 122), epigenetic-driven mechanisms (123), microRNAs (124), and miscellaneous exogenous stimuli (125).

Sex-related differences can be initially distinguished in the gene expression level in different brain areas. For instance, Berchtold and colleagues identified profound sex-based genetic changes in hippocampus and entorhinal cortex in a human cohort between 20 and 99 years of age (126). Notably, hippocampal alterations in gene expression that were detected in the aged brain were primarily originated in the complement pathway of microglial populations (117). Although there is paucity of data pertaining sex hormones and DNA repair mechanism, a system that progressively worsens due to loss of genomic maintenance in the aging brain, various investigations have linked estrogen antiaging neuroprotective mechanisms to different DNA repair enzymes (127). Additionally, epigenetic-mediated mechanisms such as immune training and tolerance lead to differential epigenetic reprogramming of microglia, as seen in a mouse model of Alzheimer's pathology, suggesting a tool to take advantage of aging (128).

Aging fosters a slow yet significant microglia dysregulation over time. A marked upregulation in female genes associated with inflammation and immune function was observed compared with the male gene pool, highlighted in ovariectomized rat experiments after studying microglia activation (129). This increase in neuroinflammation mostly seen with TNFα and IL-1β was attributed to the lack of ovarian function in aged mice (130). It is widely established that a variety of physiological and pathological events can cause activated microglia to polarize either toward a proinflammatory/cytotoxic M1 or an anti-inflammatory/neuroprotective M2 phenotype (119, 131). Growing evidence in aging and neurodegenerative diseases suggests that the polarization toward the neuroprotective status can be triggered by estrogens (132, 133), and this has been proven by estrogen replacement treatment through microglial ER subtypes, ERα and ERβ (134–136).

Clusters of genes and their relative messenger RNAs (mRNAs) that regulate microglia's sensing functions are referred to as the “sensome” (137). Those, among other genes, have been involved in microglia priming, which is an exaggerated or heightened response, yet ineffective in a senescent CNS and differs between sexes (116). The differential expression of multiple microglial genes such as Spp1, Apoe, Ccl3, Clec7a, and Ccl4 in female mice may explain sex-related differences in aging and AD (117, 138). One of the most interesting microglia mediators is Tyrobp, also known as TREM2; however, its involvement in AD is still under investigation (139). Clusters of homeostatic genes, but more importantly, another group of genes, including Ms4a7, Klra2, Clec12a, and Mrc1, were found to be upregulated in female EAE mice with single-cell sequencing (scRNA-seq) of CD45+ cells (140). The authors of this study concluded that the cells implicated in antigen presentation such as DCs and monocyte-derived microglial cells are purposeful targets during EAE.

Another age-related, female-predominant pattern of gene expression in a plethora of genes of the complement system such as C1qa, C1qc, and Ccl4 was also identified (117). Similarly, complement cascade genes and interleukin 1 receptor-like 1 (IL1RL1) were increased in women in a human cohort (141). Finally, the cellular population equally affected by age-related female traits, such as menopause, are astroglial cells, producing dystrophic astrocytes. With aging, these cells exhibit increased expression of the intermediate glial fibrillary acidic protein (GFAP) and filamentous vimentin thereby accumulating as toxic aggregates (142). Taken together, experimental and clinical studies reveal that menopause and aging both promote neuroinflammation, which may explain the sex disparities in age-related neurological diseases such as AD and MS.

### 4.2. Implications for immune-mediated neurodegeneration

As discussed thus far in neuroinflammatory conditions, which differ between males and females with age, escalating toxicity and neuronal death may contribute to neurodegeneration, however, to a lesser extent. The molecular cross-talk of autoimmunity variations and resulting neurodegeneration may be based on different imprinting of X chromosome genes, as proved by studies of T-lymphocyte DNA methylation of the X chromosome gene Foxp3, as well as by differential expression of neuronal Toll-like receptor 7 (TLR7), which is another X chromosome gene (67). A study showed that progesterone levels in the post-reproductive ages may lead to an increase in Tregs in lymphoid tissues and blood in males than females (143). CD4+CD25+ Treg and CD4+FoxP3+ Tregs functional capacity has been suggested to be enhanced with aging in men and male mice than women and female mice, respectively, because FoxP3 expression upregulates Treg functionality (95). In support of this finding, the greater potency of CD4+FoxP3+ T cells in old males than females could be correlated with changes in gonadal steroid levels as estrogens and androgens also influence FoxP3 expression (96). Conclusively, not many studies have pointed out the neurodegenerative effects of EAE; however, Tregs seem to be of significance as immune key players in the diversity of male and female neurodegeneration.

## 5. Conclusion

As with several autoimmune diseases, MS is increasingly and universally recognized to be more frequent in female than male patients. An increasing body of evidence points toward sex-specific differences in the immune system and CNS caused by effects of chromosomes, hormones, and aspects of the immune system associated with barrier function, cell migration, and effector phenotype activation of immune cellular components. Age is also a contributing factor in disease pathology and evolution, as evidenced by clinical, epidemiological, and molecular data derived from single-cell techniques, high-throughput applications, and systems immunology analyses. Several experimental models have been used in order to depict aspects of MS and/or CNS neuroinflammation, with EAE being the most widely used murine model for MS. Although EAE exhibits discrete differences from MS in terms of phenotype and pathology, it is a useful model that recapitulates specific aspects of MS evolution and thus has been extensively studied in order to offer mechanistic insights regarding the complex interaction of biological pathways implicated in the disease ontogeny, such as sex and aging processes. In this study, we summarize current knowledge stemming from EAE models regarding the effects of sex and aging on disease phenotype with relevance to the immune system and the CNS. Sex and aging associations in MS are increasingly recognized as factors determining, at least in part, disease outcomes, a knowledge with profound implications for disease management and novel treatment development.

## Author contributions

MB and NG: conceptualization. CK, EKa, EKe, MB, and PT: original drafting and writing. AA, YB, MB, NG, CK, EKa, EKe, PT, and IM: revising. NG: supervising. All authors contributed to the article and approved the submitted version.

## References

[1] Kawachi I Lassmann H. Neurodegeneration in multiple sclerosis and neuromyelitis optica. J Neurol Neurosurg Psychiatry. (2017) 88:137–45. 10.1136/jnnp-2016-313300 27671902

[2] Whitacre CC Reingold SC O'Looney PA. A gender gap in autoimmunity. Science. (1999) 283:1277–8. 10.1126/science.283.5406.1277 10084932

[3] Kingwell E Marriott JJ Jette N Pringsheim T Makhani N Morrow SA. Incidence and prevalence of multiple sclerosis in Europe: a systematic review. BMC Neurol. (2013) 13:128. 10.1186/1471-2377-13-128 24070256 PMC3856596

[4] Lopez-Otin C Blasco MA Partridge L Serrano M Kroemer G. The hallmarks of aging. Cell. (2013) 153:1194–217. 10.1016/j.cell.2013.05.039 23746838 PMC3836174

[5] Mogilenko DA Shchukina I Artyomov MN. Immune ageing at single-cell resolution. Nat Rev Immunol. (2022) 22:484–98. 10.1038/s41577-021-00646-4 34815556 PMC8609266

[6] Lassmann H Bradl M. Multiple sclerosis: experimental models and reality. Acta Neuropathol. (2017) 133:223–44. 10.1007/s00401-016-1631-4 27766432 PMC5250666

[7] Ransohoff RM. Animal models of multiple sclerosis: the good, the bad and the bottom line. Nat Neurosci. (2012) 15:1074–7. 10.1038/nn.3168 22837037 PMC7097342

[8] Koch-Henriksen N Sorensen PS. The changing demographic pattern of multiple sclerosis epidemiology. The Lancet Neurology. (2010) 9:520–32. 10.1016/S1474-4422(10)70064-8 20398859

[9] Barnett MH Williams DB Day S Macaskill P McLeod JG. Progressive increase in incidence and prevalence of multiple sclerosis in Newcastle, Australia: a 35-year study. J Neurol Sci. (2003) 213:1–6. 10.1016/S0022-510X(03)00122-9 12873746

[10] Grytten N Glad SB Aarseth JH Nyland H Midgard R Myhr KM. 50-year follow-up of the incidence of multiple sclerosis in Hordaland County, Norway. Neurology. (2006) 66:182–6. 10.1212/01.wnl.0000195549.95448.b9 16434650

[11] Orton SM Herrera BM Yee IM Valdar W Ramagopalan SV Sadovnick AD. Sex ratio of multiple sclerosis in Canada: a longitudinal study. Lancet Neurol. (2006) 5:932–6. 10.1016/S1474-4422(06)70581-6 17052660

[12] Westerlind H Bostrom I Stawiarz L Landtblom AM Almqvist C Hillert J. New data identify an increasing sex ratio of multiple sclerosis in Sweden. Mult Scler. (2014) 20:1578–83. 10.1177/1352458514530021 24842964 PMC4230455

[13] Kampman MT Aarseth JH Grytten N Benjaminsen E Celius EG Dahl OP. Sex ratio of multiple sclerosis in persons born from 1930 to 1979 and its relation to latitude in Norway. J Neurol. (2013) 260:1481–8. 10.1007/s00415-012-6814-x 23292231

[14] Trojano M Lucchese G Graziano G Taylor BV Simpson S Lepore V. Geographical variations in sex ratio trends over time in multiple sclerosis. PLoS ONE. (2012) 7:e48078. 10.1371/journal.pone.0048078 23133550 PMC3485003

[15] Hait NC Wise LE Allegood JC O'Brien M Avni D Reeves TM. Active, phosphorylated fingolimod inhibits histone deacetylases and facilitates fear extinction memory. Nat Neurosci. (2014) 17:971–80. 10.1038/nn.3728 24859201 PMC4256678

[16] Ruhrmann S Ewing E Piket E Kular L Cetrulo Lorenzi JC Fernandes SJ. Hypermethylation of MIR21 in CD4+ T cells from patients with relapsing-remitting multiple sclerosis associates with lower miRNA-21 levels and concomitant up-regulation of its target genes. Mult Scler. (2018) 24:1288–300. 10.1177/1352458517721356 28766461 PMC5794671

[17] Singhal NK Huang H Li S Clements R Gadd J Daniels A. The neuronal metabolite NAA regulates histone H3 methylation in oligodendrocytes and myelin lipid composition. Exper Brain Res. (2017) 235:279–92. 10.1007/s00221-016-4789-z 27709268 PMC5875422

[18] Keane JT Afrasiabi A Schibeci SD Fewings N Parnell GP Swaminathan S. Gender and the sex hormone estradiol affect multiple sclerosis risk gene expression in epstein-barr virus-infected B cells. Front Immunol. (2021) 12:732694. 10.3389/fimmu.2021.732694 34566997 PMC8455923

[19] Held U Heigenhauser L Shang C Kappos L Polman C. Sylvia Lawry Centre for MSR: Predictors of relapse rate in MS clinical trials. Neurology. (2005) 65:1769–73. 10.1212/01.wnl.0000187122.71735.1f 16344520

[20] Kalincik T Vivek V Jokubaitis V Lechner-Scott J Trojano M Izquierdo G. Sex as a determinant of relapse incidence and progressive course of multiple sclerosis. Brain J Neurol. (2013) 136:3609s−17. 10.1093/brain/awt281 24142147

[21] Magyari M Koch-Henriksen N. Quantitative effect of sex on disease activity and disability accumulation in multiple sclerosis. J Neurol Neurosurg Psychiatry. (2022) 93:716–22. 10.1136/jnnp-2022-328994 35393340 PMC9279846

[22] Ribbons KA McElduff P Boz C Trojano M Izquierdo G Duquette P. Male sex is independently associated with faster disability accumulation in relapse-onset ms but not in primary progressive MS. PLoS ONE. (2015) 10:e0122686. 10.1371/journal.pone.0122686 26046348 PMC4457630

[23] Bove R Okai A Houtchens M Elias-Hamp B Lugaresi A Hellwig K. Effects of menopause in women with multiple sclerosis: an evidence-based review. Front Neurol. (2021) 12:554375. 10.3389/fneur.2021.554375 33815241 PMC8017266

[24] Bove R Healy BC Musallam A Glanz BI De Jager PL Chitnis T. Exploration of changes in disability after menopause in a longitudinal multiple sclerosis cohort. Mult Scler. (2016) 22:935–43. 10.1177/1352458515606211 26447063 PMC4824677

[25] Willer CJ Dyment DA Risch NJ Sadovnick AD Ebers GC Canadian Collaborative Study G. Twin concordance and sibling recurrence rates in multiple sclerosis. Proc Natl Acad Sci U S A. (2003) 100:12877–82. 10.1073/pnas.1932604100 14569025 PMC240712

[26] Koch M Kingwell E Rieckmann P Tremlett H Neurologists UMC. The natural history of secondary progressive multiple sclerosis. J Neurol Neurosurg Psychiatry. (2010) 81:1039–43. 10.1136/jnnp.2010.208173 20639385

[27] International Multiple Sclerosis Genetics C Wellcome Trust Case Control C Sawcer S Hellenthal G Pirinen M Spencer CC. Genetic risk and a primary role for cell-mediated immune mechanisms in multiple sclerosis. Nature. (2011) 476:214–9. 10.1038/nature10251 21833088 PMC3182531

[28] Ebers GC Sadovnick AD Dyment DA Yee IM Willer CJ Risch N. Parent-of-origin effect in multiple sclerosis: observations in half-siblings. Lancet. (2004) 363:1773–4. 10.1016/S0140-6736(04)16304-6 15172777

[29] Ma Q Caillier SJ Muzic S University of California San Francisco MSET Wilson MR Henry RG. Specific hypomethylation programs underpin B cell activation in early multiple sclerosis. Proc Natl Acad Sci U S A. (2021) 118:e2111920118. 10.1073/pnas.2111920118 34911760 PMC8713784

[30] Ramagopalan SV Herrera BM Bell JT Dyment DA Deluca GC Lincoln MR. Parental transmission of HLA-DRB1^*^15 in multiple sclerosis. Hum Genet. (2008) 122:661–3. 10.1007/s00439-007-0442-z 17972102

[31] International Multiple Sclerosis Genetics C Beecham AH Patsopoulos NA Xifara DK Davis MF Kemppinen A. Analysis of immune-related loci identifies 48 new susceptibility variants for multiple sclerosis. Nat Genet. (2013) 45:1353–60. 10.1038/ng.2770 24076602 PMC3832895

[32] Briggs FB Acuna B Shen L Ramsay P Quach H Bernstein A. Smoking and risk of multiple sclerosis: evidence of modification by NAT1 variants. Epidemiology. (2014) 25:605–14. 10.1097/EDE.0000000000000089 24625537

[33] Hedstrom AK Sundqvist E Baarnhielm M Nordin N Hillert J Kockum I. Smoking and two human leukocyte antigen genes interact to increase the risk for multiple sclerosis. Brain. (2011) 134:653–64. 10.1093/brain/awq371 21303861

[34] Maltby VE Lea RA Graves MC Sanders KA Benton MC Tajouri L. Genome-wide DNA methylation changes in CD19(+) B cells from relapsing-remitting multiple sclerosis patients. Sci Rep. (2018) 8:17418. 10.1038/s41598-018-35603-0 30479356 PMC6258668

[35] Kiselev I Danilova L Baulina N Baturina O Kabilov M Boyko A. Genome-wide DNA methylation profiling identifies epigenetic changes in CD4+ and CD14+ cells of multiple sclerosis patients. Mult Scler Relat Disord. (2022) 60:103714. 10.1016/j.msard.2022.103714 35245816

[36] Shen S Sandoval J Swiss VA Li J Dupree J Franklin RJ. Age-dependent epigenetic control of differentiation inhibitors is critical for remyelination efficiency. Nat Neurosci. (2008) 11:1024–34. 10.1038/nn.2172 19160500 PMC2656679

[37] Graves MC Benton M Lea RA Boyle M Tajouri L Macartney-Coxson D. Methylation differences at the HLA-DRB1 locus in CD4+ T-Cells are associated with multiple sclerosis. Mult Scler. (2014) 20:1033–41. 10.1177/1352458513516529 24336351

[38] Kular L Klose D Urdanoz-Casado A Ewing E Planell N Gomez-Cabrero D. Epigenetic clock indicates accelerated aging in glial cells of progressive multiple sclerosis patients. Front Aging Neurosci. (2022) 14:926468. 10.3389/fnagi.2022.926468 36092807 PMC9454196

[39] Zhang FF Cardarelli R Carroll J Fulda KG Kaur M Gonzalez K. Significant differences in global genomic DNA methylation by gender and race/ethnicity in peripheral blood. Epigenetics. (2011) 6:623–9. 10.4161/epi.6.5.15335 21739720 PMC3230547

[40] Benkert P Meier S Schaedelin S Manouchehrinia A Yaldizli O Maceski A. Serum neurofilament light chain for individual prognostication of disease activity in people with multiple sclerosis: a retrospective modelling and validation study. Lancet Neurol. (2022) 21:246–57. 10.1016/S1474-4422(22)00009-6 35182510

[41] Zheleznyakova GY Piket E Marabita F Pahlevan Kakhki M Ewing E Ruhrmann S. Epigenetic research in multiple sclerosis: progress, challenges, and opportunities. Physiol Genomics. (2017) 49:447–61. 10.1152/physiolgenomics.00060.2017 28754822

[42] Constantinescu CS Farooqi N O'Brien K Gran B. Experimental autoimmune encephalomyelitis (EAE) as a model for multiple sclerosis (MS). Br J Pharmacol. (2011) 164:1079–106. 10.1111/j.1476-5381.2011.01302.x 21371012 PMC3229753

[43] Robinson AP Harp CT Noronha A Miller SD. The experimental autoimmune encephalomyelitis (EAE) model of MS: utility for understanding disease pathophysiology and treatment. Handb Clin Neurol. (2014) 122:173–89. 10.1016/B978-0-444-52001-2.00008-X 24507518 PMC3981554

[44] Theotokis P Kesidou E Mitsiadou D Petratos S Damianidou O Boziki M. Lumbar spine intrathecal transplantation of neural precursor cells promotes oligodendrocyte proliferation in hot spots of chronic demyelination. Brain Pathol. (2022) 32:e13040. 10.1111/bpa.13040 34845781 PMC9245942

[45] Steinman L Zamvil SS. How to successfully apply animal studies in experimental allergic encephalomyelitis to research on multiple sclerosis. Ann Neurol. (2006) 60:12–21. 10.1002/ana.20913 16802293

[46] Kipp M Nyamoya S Hochstrasser T Amor S. Multiple sclerosis animal models: a clinical and histopathological perspective. Brain Pathol. (2017) 27:123–37. 10.1111/bpa.12454 27792289 PMC8029141

[47] Stromnes IM Goverman JM. Passive induction of experimental allergic encephalomyelitis. Nat Protoc. (2006) 1:1952–60. 10.1038/nprot.2006.284 17487182

[48] Bettelli E. Building different mouse models for human MS. Ann N Y Acad Sci. (2007) 1103:11–8. 10.1196/annals.1394.021 17376825

[49] McRae BL Kennedy MK Tan LJ Dal Canto MC Picha KS Miller SD. Induction of active and adoptive relapsing experimental autoimmune encephalomyelitis (EAE) using an encephalitogenic epitope of proteolipid protein. J Neuroimmunol. (1992) 38:229–40. 10.1016/0165-5728(92)90016-E 1376328

[50] Sobel RA Greer JM Kuchroo VK. Minireview: autoimmune responses to myelin proteolipid protein. Neurochem Res. (1994) 19:915–21. 10.1007/BF00968701 7528354

[51] Mendel I Kerlero de Rosbo N Ben-Nun A. A myelin oligodendrocyte glycoprotein peptide induces typical chronic experimental autoimmune encephalomyelitis in H-2b mice: fine specificity and T cell receptor V beta expression of encephalitogenic T cells. Eur J Immunol. (1995) 25:1951–9. 10.1002/eji.1830250723 7621871

[52] Hampton DW Serio A Pryce G Al-Izki S Franklin RJ Giovannoni G. Neurodegeneration progresses despite complete elimination of clinical relapses in a mouse model of multiple sclerosis. Acta Neuropathol Commun. (2013) 1:84. 10.1186/2051-5960-1-84 24364862 PMC3895761

[53] Mony JT Khorooshi R Owens T MOG. extracellular domain (p1-125) triggers elevated frequency of CXCR3+ CD4+ Th1 cells in the CNS of mice and induces greater incidence of severe EAE. Mult Scler. (2014) 20:1312–21. 10.1177/1352458514524086 24552747

[54] Theotokis P Lourbopoulos A Touloumi O Lagoudaki R Kofidou E Nousiopoulou E. Time course and spatial profile of Nogo-A expression in experimental autoimmune encephalomyelitis in C57BL/6 mice. J Neuropathol Exp Neurol. (2012) 71:907–20. 10.1097/NEN.0b013e31826caebe 22964785

[55] Theotokis P Kleopa KA Touloumi O Lagoudaki R Lourbopoulos A Nousiopoulou E. Connexin43 and connexin47 alterations after neural precursor cells transplantation in experimental autoimmune encephalomyelitis. Glia. (2015) 63:1772–83. 10.1002/glia.22843 25914045

[56] Krishnamoorthy G Wekerle H EAE. an immunologist's magic eye. Eur J Immunol. (2009) 39:2031–5. 10.1002/eji.200939568 19672898

[57] Pollinger B Krishnamoorthy G Berer K Lassmann H Bosl MR Dunn R. Spontaneous relapsing-remitting EAE in the SJL/J mouse: MOG-reactive transgenic T cells recruit endogenous MOG-specific B cells. J Exp Med. (2009) 206:1303–16. 10.1084/jem.20090299 19487416 PMC2715069

[58] Umair M Fazazi MR Rangachari M. Biological Sex As a Critical Variable in CD4(+) Effector T Cell Function in Preclinical Models of Multiple Sclerosis. Antioxid Redox Signal. (2022) 37:135–49. 10.1089/ars.2021.0202 34538129 PMC9293683

[59] Papenfuss TL Rogers CJ Gienapp I Yurrita M McClain M Damico N. Sex differences in experimental autoimmune encephalomyelitis in multiple murine strains. J Neuroimmunol. (2004) 150:59–69. 10.1016/j.jneuroim.2004.01.018 15081249

[60] Bebo BF Zelinka-Vincent E Adamus G Amundson D Vandenbark AA Offner H. Gonadal hormones influence the immune response to PLP 139-151 and the clinical course of relapsing experimental autoimmune encephalomyelitis. J Neuroimmunol. (1998) 84:122–30. 10.1016/S0165-5728(97)00214-2 9628453

[61] Dalal M Kim S Voskuhl RR. Testosterone therapy ameliorates experimental autoimmune encephalomyelitis and induces a T helper 2 bias in the autoantigen-specific T lymphocyte response. J Immunol. (1997) 159:3–6. 9200430

[62] Bebo BF Schuster JC Vandenbark AA Offner H. Androgens alter the cytokine profile and reduce encephalitogenicity of myelin-reactive T cells. J Immunol. (1999) 162:35–40. 9886367

[63] Arnold AP Chen X. What does the “four core genotypes” mouse model tell us about sex differences in the brain and other tissues? Front Neuroendocrinol. (2009) 30:1–9. 10.1016/j.yfrne.2008.11.001 19028515 PMC3282561

[64] Cox KH Bonthuis PJ Rissman EF. Mouse model systems to study sex chromosome genes and behavior: relevance to humans. Front Neuroendocrinol. (2014) 35:405–19. 10.1016/j.yfrne.2013.12.004 24388960 PMC4079771

[65] Itoh Y Mackie R Kampf K Domadia S Brown JD O'Neill R. Four core genotypes mouse model: localization of the Sry transgene and bioassay for testicular hormone levels. BMC Res Notes. (2015) 8:69. 10.1186/s13104-015-0986-2 25870930 PMC4354741

[66] Arnold AP. Four Core Genotypes and XY^*^ mouse models: Update on impact on SABV research. Neurosci Biobehav Rev. (2020) 119:1–8. 10.1016/j.neubiorev.2020.09.021 32980399 PMC7736196

[67] Voskuhl RR Sawalha AH Itoh Y. Sex chromosome contributions to sex differences in multiple sclerosis susceptibility and progression. Mult Scler. (2018) 24:22–31. 10.1177/1352458517737394 29307297 PMC5823689

[68] Smith-Bouvier DL Divekar AA Sasidhar M Du S Tiwari-Woodruff SK King JK. A role for sex chromosome complement in the female bias in autoimmune disease. J Exp Med. (2008) 205:1099–108. 10.1084/jem.20070850 18443225 PMC2373842

[69] Du S Itoh N Askarinam S Hill H Arnold AP Voskuhl RR. sex chromosome complement, compared with XX, in the CNS confers greater neurodegeneration during experimental autoimmune encephalomyelitis. Proc Natl Acad Sci U S A. (2014) 111:2806–11. 10.1073/pnas.1307091111 24550311 PMC3932937

[70] Teuscher C Noubade R Spach K McElvany B Bunn JY Fillmore PD. Evidence that the Y chromosome influences autoimmune disease in male and female mice. Proc Natl Acad Sci U S A. (2006) 103:8024–9. 10.1073/pnas.0600536103 16702550 PMC1472423

[71] Butterfield RJ Blankenhorn EP Roper RJ Zachary JF Doerge RW Sudweeks J. Genetic analysis of disease subtypes and sexual dimorphisms in mouse experimental allergic encephalomyelitis (EAE): relapsing/remitting and monophasic remitting/nonrelapsing EAE are immunogenetically distinct. J Immunol. (1999) 162:3096–102. 10072563

[72] Jiang PP Frederick K Hansen TH Miller RD. Localization of the mouse gene releasing sex-limited expression of Slp. Proc Natl Acad Sci U S A. (1996) 93:913–7. 10.1073/pnas.93.2.913 8570659 PMC40158

[73] Lasrado N Jia T Massilamany C Franco R Illes Z Reddy J. Mechanisms of sex hormones in autoimmunity: focus on EAE. Biol Sex Differ. (2020) 11:50. 10.1186/s13293-020-00325-4 32894183 PMC7475723

[74] Paharkova-Vatchkova V Maldonado R Kovats S. Estrogen preferentially promotes the differentiation of CD11c+ CD11b(intermediate) dendritic cells from bone marrow precursors. J Immunol. (2004) 172:1426–36. 10.4049/jimmunol.172.3.1426 14734718

[75] Fitzpatrick FT Greenstein BD. Effects of various steroids on the thymus, spleen, ventral prostate and seminal vesicles in old orchidectomized rats. J Endocrinol. (1987) 113:51–5. 10.1677/joe.0.1130051 3585226

[76] Okasha SA Ryu S Do Y McKallip RJ Nagarkatti M Nagarkatti PS. Evidence for estradiol-induced apoptosis and dysregulated T cell maturation in the thymus. Toxicology. (2001) 163:49–62. 10.1016/S0300-483X(01)00374-2 11376864

[77] Wang C Dehghani B Magrisso IJ Rick EA Bonhomme E Cody DB. GPR30 contributes to estrogen-induced thymic atrophy. Mol Endocrinol. (2008) 22:636–48. 10.1210/me.2007-0359 18063692 PMC2262170

[78] Do Y Ryu S Nagarkatti M Nagarkatti PS. Role of death receptor pathway in estradiol-induced T-cell apoptosis in vivo. Toxicol Sci. (2002) 70:63–72. 10.1093/toxsci/70.1.63 12388836

[79] Lindberg MK Weihua Z Andersson N Moverare S Gao H Vidal O. Estrogen receptor specificity for the effects of estrogen in ovariectomized mice. J Endocrinol. (2002) 174:167–78. 10.1677/joe.0.1740167 12176656

[80] Zoller AL Schnell FJ Kersh GJ. Murine pregnancy leads to reduced proliferation of maternal thymocytes and decreased thymic emigration. Immunology. (2007) 121:207–15. 10.1111/j.1365-2567.2006.02559.x 17250584 PMC2265940

[81] Zoller AL Kersh GJ. Estrogen induces thymic atrophy by eliminating early thymic progenitors and inhibiting proliferation of beta-selected thymocytes. J Immunol. (2006) 176:7371–8. 10.4049/jimmunol.176.12.7371 16751381

[82] Seifert HA Benedek G Nguyen H Kent G Vandenbark AA Offner H. Estrogen protects both sexes against EAE by promoting common regulatory cell subtypes independent of endogenous estrogen. Metab Brain Dis. (2017) 32:1747–54. 10.1007/s11011-017-0063-8 28689297 PMC5650507

[83] Bebo BF Fyfe-Johnson A Adlard K Beam AG Vandenbark AA Offner H. Low-dose estrogen therapy ameliorates experimental autoimmune encephalomyelitis in two different inbred mouse strains. J Immunol. (2001) 166:2080–9. 10.4049/jimmunol.166.3.2080 11160259

[84] Ito A Bebo BF Matejuk A Zamora A Silverman M Fyfe-Johnson A. Estrogen treatment down-regulates TNF-alpha production and reduces the severity of experimental autoimmune encephalomyelitis in cytokine knockout mice. J Immunol. (2001) 167:542–52. 10.4049/jimmunol.167.1.542 11418693

[85] Liu HY Buenafe AC Matejuk A Ito A Zamora A Dwyer J. Estrogen inhibition of EAE involves effects on dendritic cell function. J Neurosci Res. (2002) 70:238–48. 10.1002/jnr.10409 12271473

[86] Polanczyk M Zamora A Subramanian S Matejuk A Hess DL Blankenhorn EP. The protective effect of 17beta-estradiol on experimental autoimmune encephalomyelitis is mediated through estrogen receptor-alpha. Am J Pathol. (2003) 163:1599–605. 10.1016/S0002-9440(10)63516-X 14507666 PMC1868300

[87] Kim S Liva SM Dalal MA Verity MA Voskuhl RR. Estriol ameliorates autoimmune demyelinating disease: implications for multiple sclerosis. Neurology. (1999) 52:1230–8. 10.1212/WNL.52.6.1230 10214749

[88] Liu HB Loo KK Palaszynski K Ashouri J Lubahn DB Voskuhl RR. Estrogen receptor alpha mediates estrogen's immune protection in autoimmune disease. J Immunol. (2003) 171:6936–40. 10.4049/jimmunol.171.12.6936 14662901

[89] Palaszynski KM Liu H Loo KK Voskuhl RR. Estriol treatment ameliorates disease in males with experimental autoimmune encephalomyelitis: implications for multiple sclerosis. J Neuroimmunol. (2004) 149:84–9. 10.1016/j.jneuroim.2003.12.015 15020068

[90] Subramanian S Matejuk A Zamora A Vandenbark AA Offner H. Oral feeding with ethinyl estradiol suppresses and treats experimental autoimmune encephalomyelitis in SJL mice and inhibits the recruitment of inflammatory cells into the central nervous system. J Immunol. (2003) 170:1548–55. 10.4049/jimmunol.170.3.1548 12538720

[91] Garcia-Segura LM Naftolin F Hutchison JB Azcoitia I Chowen JA. Role of astroglia in estrogen regulation of synaptic plasticity and brain repair. J Neurobiol. (1999) 40:574–84. 10.1002/(SICI)1097-4695(19990915)40:4<574::AID-NEU12>3.0.CO;2-8 10453057

[92] Bruce-Keller AJ Keeling JL Keller JN Huang FF Camondola S Mattson MP. Antiinflammatory effects of estrogen on microglial activation. Endocrinology. (2000) 141:3646–56. 10.1210/endo.141.10.7693 11014219

[93] Morales LB Loo KK Liu HB Peterson C Tiwari-Woodruff S Voskuhl RR. Treatment with an estrogen receptor alpha ligand is neuroprotective in experimental autoimmune encephalomyelitis. J Neurosci. (2006) 26:6823–33. 10.1523/JNEUROSCI.0453-06.2006 16793889 PMC6673842

[94] Polanczyk MJ Hopke C Vandenbark AA Offner H. Treg suppressive activity involves estrogen-dependent expression of programmed death-1 (PD-1). Int Immunol. (2007) 19:337–43. 10.1093/intimm/dxl151 17267414

[95] Rosenkranz D Weyer S Tolosa E Gaenslen A Berg D Leyhe T. Higher frequency of regulatory T cells in the elderly and increased suppressive activity in neurodegeneration. J Neuroimmunol. (2007) 188:117–27. 10.1016/j.jneuroim.2007.05.011 17582512

[96] Nie J Li YY Zheng SG Tsun A Li B. FOXP3(+) Treg cells and gender bias in autoimmune diseases. Front Immunol. (2015) 6:493. 10.3389/fimmu.2015.00493 26441996 PMC4585344

[97] Mathis D Benoist C. A decade of AIRE. Nat Rev Immunol. (2007) 7:645–50. 10.1038/nri2136 17641664

[98] Medina KL Smithson G Kincade PW. Suppression of B lymphopoiesis during normal pregnancy. J Exp Med. (1993) 178:1507–15. 10.1084/jem.178.5.1507 8228804 PMC2191236

[99] Benedek G Chaudhary P Meza-Romero R Calkins E Kent G Offner H. Sex-dependent treatment of chronic EAE with partial MHC class II constructs. J Neuroinflammation. (2017) 14:100. 10.1186/s12974-017-0873-y 28477623 PMC5420407

[100] Benedek G Meza-Romero R Jordan K Keenlyside L Offner H Vandenbark AA. HLA-DRalpha1-mMOG-35-55 treatment of experimental autoimmune encephalomyelitis reduces CNS inflammation, enhances M2 macrophage frequency, and promotes neuroprotection. J Neuroinflammation. (2015) 12:123. 10.1186/s12974-015-0342-4 26104759 PMC4481122

[101] Wang C Gold BG Kaler LJ Yu X Afentoulis ME Burrows GG. Antigen-specific therapy promotes repair of myelin and axonal damage in established EAE. J Neurochem. (2006) 98:1817–27. 10.1111/j.1471-4159.2006.04081.x 16899071 PMC2175524

[102] Liva SM Voskuhl RR. Testosterone acts directly on CD4+ T lymphocytes to increase IL-10 production. J Immunol. (2001) 167:2060–7. 10.4049/jimmunol.167.4.2060 11489988

[103] Ziehn MO Avedisian AA Dervin SM Umeda EA O'Dell TJ Voskuhl RR. Therapeutic testosterone administration preserves excitatory synaptic transmission in the hippocampus during autoimmune demyelinating disease. J Neurosci. (2012) 32:12312–24. 10.1523/JNEUROSCI.2796-12.2012 22956822 PMC3571760

[104] Trapp BD Wujek JR Criste GA Jalabi W Yin X Kidd GJ. Evidence for synaptic stripping by cortical microglia. Glia. (2007) 55:360–8. 10.1002/glia.20462 17136771

[105] Weber CM Clyne AM. Sex differences in the blood-brain barrier and neurodegenerative diseases. APL Bioeng. (2021) 5:011509. 10.1063/5.0035610 33758788 PMC7968933

[106] Maggioli E McArthur S Mauro C Kieswich J Kusters DHM Reutelingsperger CPM. Estrogen protects the blood-brain barrier from inflammation-induced disruption and increased lymphocyte trafficking. Brain Behav Immun. (2016) 51:212–22. 10.1016/j.bbi.2015.08.020 26321046

[107] Batzdorf CS Morr AS Bertalan G Sack I Silva RV Infante-Duarte C. Sexual dimorphism in extracellular matrix composition and viscoelasticity of the healthy and inflamed mouse brain. Biology. (2022) 11:230. 10.3390/biology11020230 35205095 PMC8869215

[108] Park HJ Choi JM. Sex-specific regulation of immune responses by PPARs. Exp Mol Med. (2017) 49:e364. 10.1038/emm.2017.102 28775365 PMC5579504

[109] Dunn SE Ousman SS Sobel RA Zuniga L Baranzini SE Youssef S. Peroxisome proliferator-activated receptor (PPAR)alpha expression in T cells mediates gender differences in development of T cell-mediated autoimmunity. J Exp Med. (2007) 204:321–30. 10.1084/jem.20061839 17261635 PMC2118721

[110] Wiedrick J Meza-Romero R Gerstner G Seifert H Chaudhary P Headrick A. Sex differences in EAE reveal common and distinct cellular and molecular components. Cell Immunol. (2021) 359:104242. 10.1016/j.cellimm.2020.104242 33190849 PMC7770093

[111] Han J Zhu K Zhou K Hakim R Sankavaram SR Blomgren K. Sex-specific effects of microglia-like cell engraftment during experimental autoimmune encephalomyelitis. Int J Mol Sci. (2020) 21:6824. 10.3390/ijms21186824 32957621 PMC7555782

[112] Tassoni A Farkhondeh V Itoh Y Itoh N Sofroniew MV Voskuhl RR. The astrocyte transcriptome in EAE optic neuritis shows complement activation and reveals a sex difference in astrocytic C3 expression. Sci Rep. (2019) 9:10010. 10.1038/s41598-019-46232-6 31292459 PMC6620300

[113] Marrocco J McEwen BS. Sex in the brain: hormones and sex differences. Dialogues Clin Neurosci. (2016) 18:373–83. 10.31887/DCNS.2016.18.4/jmarrocco 28179809 PMC5286723

[114] Miclea A Salmen A Zoehner G Diem L Kamm CP Chaloulos-Iakovidis P. Age-dependent variation of female preponderance across different phenotypes of multiple sclerosis: A retrospective cross-sectional study. CNS Neurosci Ther. (2019) 25:527–31. 10.1111/cns.13083 30411534 PMC6488902

[115] Barron AM Pike CJ. Sex hormones, aging, and Alzheimer's disease. Front Biosci (Elite Ed). (2012) 4:976–97. 10.2741/e434 22201929 PMC3511049

[116] Nissen JC. Microglial function across the spectrum of age and gender. Int J Mol Sci. (2017) 18:561. 10.3390/ijms18030561 28273860 PMC5372577

[117] Mangold CA Wronowski B Du M Masser DR Hadad N Bixler GV. Sexually divergent induction of microglial-associated neuroinflammation with hippocampal aging. J Neuroinflammation. (2017) 14:141. 10.1186/s12974-017-0920-8 28732515 PMC5521082

[118] Acosta-Martinez M. Shaping Microglial Phenotypes Through Estrogen Receptors: Relevance to Sex-Specific Neuroinflammatory Responses to Brain Injury and Disease. J Pharmacol Exp Ther. (2020) 375:223–36. 10.1124/jpet.119.264598 32513838

[119] Villa A Vegeto E Poletti A Maggi A. Estrogens, Neuroinflammation, and Neurodegeneration. Endocr Rev. (2016) 37:372–402. 10.1210/er.2016-1007 27196727 PMC4971309

[120] Lenz KM Nelson LH. Microglia and beyond: innate immune cells as regulators of brain development and behavioral function. Front Immunol. (2018) 9:698. 10.3389/fimmu.2018.00698 29706957 PMC5908908

[121] Klein SL Flanagan KL. Sex differences in immune responses. Nat Rev Immunol. (2016) 16:626–38. 10.1038/nri.2016.90 27546235

[122] Lefevre N Corazza F Valsamis J Delbaere A De Maertelaer V Duchateau J. The number of X chromosomes influences inflammatory cytokine production following toll-like receptor stimulation. Front Immunol. (2019) 10:1052. 10.3389/fimmu.2019.01052 31143188 PMC6521177

[123] Cheray M Joseph B. Epigenetics control microglia plasticity. Front Cell Neurosci. (2018) 12:243. 10.3389/fncel.2018.00243 30123114 PMC6085560

[124] Kodama L Guzman E Etchegaray JI Li Y Sayed FA Zhou L. Microglial microRNAs mediate sex-specific responses to tau pathology. Nat Neurosci. (2020) 23:167–71. 10.1038/s41593-019-0560-7 31873194 PMC7394069

[125] Lynch MA. Exploring sex-related differences in microglia may be a game-changer in precision medicine. Front Aging Neurosci. (2022) 14:868448. 10.3389/fnagi.2022.868448 35431903 PMC9009390

[126] Berchtold NC Cribbs DH Coleman PD Rogers J Head E Kim R. Gene expression changes in the course of normal brain aging are sexually dimorphic. Proc Natl Acad Sci U S A. (2008) 105:15605–10. 10.1073/pnas.0806883105 18832152 PMC2563070

[127] Zarate S Stevnsner T Gredilla R. Role of estrogen and other sex hormones in brain aging: Neuroprotection and DNA repair. Front Aging Neurosci. (2017) 9:430. 10.3389/fnagi.2017.00430 29311911 PMC5743731

[128] Wendeln AC Degenhardt K Kaurani L Gertig M Ulas T Jain G. Innate immune memory in the brain shapes neurological disease hallmarks. Nature. (2018) 556:332–8. 10.1038/s41586-018-0023-4 29643512 PMC6038912

[129] Sarvari M Hrabovszky E Kallo I Solymosi N Liko I Berchtold N. Menopause leads to elevated expression of macrophage-associated genes in the aging frontal cortex: rat and human studies identify strikingly similar changes. J Neuroinflammation. (2012) 9:264. 10.1186/1742-2094-9-264 23206327 PMC3558453

[130] Benedusi V Meda C Della Torre S Monteleone G Vegeto E Maggi A. lack of ovarian function increases neuroinflammation in aged mice. Endocrinology. (2012) 153:2777–88. 10.1210/en.2011-1925 22492304 PMC3359599

[131] Labandeira-Garcia JL Rodriguez-Perez AI Garrido-Gil P Rodriguez-Pallares J Lanciego JL Guerra MJ. Brain renin-angiotensin system and microglial polarization: implications for aging and neurodegeneration. Front Aging Neurosci. (2017) 9:129. 10.3389/fnagi.2017.00129 28515690 PMC5413566

[132] Siani F Greco R Levandis G Ghezzi C Daviddi F Demartini C. Influence of estrogen modulation on glia activation in a murine model of Parkinson's disease. Front Neurosci. (2017) 11:306. 10.3389/fnins.2017.00306 28620274 PMC5449471

[133] Selvamani A Sohrabji F. Reproductive age modulates the impact of focal ischemia on the forebrain as well as the effects of estrogen treatment in female rats. Neurobiol Aging. (2010) 31:1618–28. 10.1016/j.neurobiolaging.2008.08.014 18829137 PMC2909345

[134] Sarvari M Kallo I Hrabovszky E Solymosi N Rodolosse A Liposits Z. Long-term estrogen receptor beta agonist treatment modifies the hippocampal transcriptome in middle-aged ovariectomized rats. Front Cell Neurosci. (2016) 10:149. 10.3389/fncel.2016.00149 27375434 PMC4901073

[135] Sarvari M Kallo I Hrabovszky E Solymosi N Rodolosse A Vastagh C. Hippocampal gene expression is highly responsive to estradiol replacement in middle-aged female rats. Endocrinology. (2015) 156:2632–45. 10.1210/en.2015-1109 25924104

[136] Sarvari M Deli L Kocsis P Mark L Maasz G Hrabovszky E. Estradiol and isotype-selective estrogen receptor agonists modulate the mesocortical dopaminergic system in gonadectomized female rats. Brain Res. (2014) 1583:1–11. 10.1016/j.brainres.2014.06.020 24976584

[137] Hickman SE Kingery ND Ohsumi TK Borowsky ML Wang LC Means TK. The microglial sensome revealed by direct RNA sequencing. Nat Neurosci. (2013) 16:1896–905. 10.1038/nn.3554 24162652 PMC3840123

[138] Kang SS Ebbert MTW Baker KE Cook C Wang X Sens JP. Microglial translational profiling reveals a convergent APOE pathway from aging, amyloid, and tau. J Exp Med. (2018) 215:2235–45. 10.1084/jem.20180653 30082275 PMC6122978

[139] Ma J Jiang T Tan L Yu JT. TYROBP in Alzheimer's disease. Mol Neurobiol. (2015) 51:820–6. 10.1007/s12035-014-8811-9 25052481

[140] Jordao MJC Sankowski R Brendecke SM Sagar Locatelli G Tai YH. Single-cell profiling identifies myeloid cell subsets with distinct fates during neuroinflammation. Science. (2019) 363:eaat7554. 10.1126/science.aat7554 30679343

[141] Trabzuni D Ramasamy A Imran S Walker R Smith C Weale ME. Widespread sex differences in gene expression and splicing in the adult human brain. Nat Commun. (2013) 4:2771. 10.1038/ncomms3771 24264146 PMC3868224

[142] Salminen A Ojala J Kaarniranta K Haapasalo A Hiltunen M Soininen H. Astrocytes in the aging brain express characteristics of senescence-associated secretory phenotype. Eur J Neurosci. (2011) 34:3–11. 10.1111/j.1460-9568.2011.07738.x 21649759

[143] Arsenovic-Ranin N Kosec D Nacka-Aleksic M Pilipovic I Stojic-Vukanic Z Djikic J. Ovarian hormone level alterations during rat post-reproductive life-span influence CD8 + T-cell homeostasis. Exp Biol Med. (2015) 240:1319–32. 10.1177/1535370215570817 25716018 PMC4935252

